# Patient and Provider Emergency Care Experiences Related to Intimate Partner Violence: A Systematic Review of the Existing Evidence

**DOI:** 10.1177/15248380221118962

**Published:** 2022-08-23

**Authors:** Emma Duchesne, Aisha Nathoo, Melanie Walker, Susan A. Bartels

**Affiliations:** 1 Queen’s University, Kingston, ON, Canada

**Keywords:** anything related to domestic violence, domestic violence and cultural contexts, perceptions of domestic violence, vicarious trauma, intimate partner violence, emergency medicine

## Abstract

Intimate partner violence (IPV) is a public health problem that has devastating physical, psychological, and economic consequences. The emergency department (ED) is an important point of contact for individuals experiencing IPV. However, there are few studies synthesizing interactions between patients experiencing IPV and providers. We aimed to summarize the existing evidence regarding (1) ED care experiences of patients with a history of IPV and (2) experiences of ED providers interacting with them. The secondary aim of this review was to evaluate high-quality care barriers and facilitators and to elucidate common causes of care avoidance. A literature search of peer-reviewed electronic databases was undertaken. Inclusion criteria consisted of studies detailing IPV-related patient or provider experiences surrounding ED visits. Articles published before 2000 or unavailable in English/French were excluded. A total of 772 studies were screened, yielding a final number of 41 studies. Negative patient experiences arose from individual-, institutional-, and system-level issues, commonly including adverse provider behavior. Negative provider experiences stemmed from individual-, institutional-, and system-level issues, such as a lack of knowledge and lack of infrastructure. Facilitators to positive patient experiences included interacting with empathetic providers, having privacy, and receiving timely specialized care. Facilitators to positive provider experiences included feeling well-equipped to manage IPV and having policies leading to appropriate care. Negative ED care experiences reveal inadequate care quality, ultimately leading to secondary victimization of individuals experiencing IPV. This review also uncovered important literature gaps regarding experiences of those who identify as equity-deserving.

## Introduction

One in three women and one in four men have experienced intimate partner violence (IPV) in their lifetime (Black et al., 2011) and, in the United States, one out of five homicide victims were killed by an intimate partner (Smith & Cooper, 2011). IPV is defined as any behavior that causes physical, psychological, or sexual harm to those within an intimate relationship and continues to be an ongoing human’s rights issue (World Health Organization, 2012). Fifty percent of women seen in emergency departments (EDs) report a history of IPV, and approximately 44% of those killed by their abuser were seen in the ED in the 2 years before their murder (Huecker et al., 2021). The ED thus represents a unique clinical environment to reduce IPV-related morbidity and mortality.

Emergency providers commonly experience frustration, limited time, and powerlessness when caring for individuals who have experienced IPV (Williston & Lafreniere, 2013). Furthermore, many patients describe ED providers as rushed and lacking compassion. This often results in negative care experiences (Yam, 2000).

Patient care experiences relate to the contact patients have with the healthcare system such as effective communication with clinicians or interactions with professional providers (Agency for Healthcare Research and Quality, 2020; Agency for Healthcare Research and Quality, 2022). Provider care experiences relate to clinician engagement in their day-to-day work, for example workflow efficiency or experiences regarding effective scheduling (Healthbox, 2019).

Patient care avoidance is described as turning away from threat-related cues, which results in not being able or willing to be involved in necessary care (Klop et al., 2018), negatively influencing an individuals’ well-being (Ye et al., 2012). Negative patient care experiences have been linked to patient care avoidance and generate negative patient outcomes (Schwei et al., 2017). Conversely, positive patient care experiences create better clinical outcomes, improved patient adherence to medical advice, better disease prevention, improved patient safety practices, and lower utilization of unnecessary healthcare services (Agency for Healthcare Research and Quality, 2022; Kelley et al., 2014).

There are few systematic reviews (SRs) delineating negative experiences from both patient and provider perspectives related to IPV care within the ED. We, therefore, conducted an SR with a primary aim of summarizing the existing evidence regarding (1) ED care experiences of patients with a history of IPV and (2) experiences of ED providers interacting with them. The secondary aim was to evaluate contributors to positive and negative care experiences, as well as uncover reasons behind patient care avoidance.

Contributors to positive and negative care were organized into three levels: individual (related to the person such as interpersonal interactions or individual practices), institutional (related to the ED such as departmental practices, norms, and infrastructure) and systemic (related to the system, including inter-institutional, historical, economic and cultural policies or norms) issues.

## Methods

We conducted a systematic literature review of health services responses to IPV, following the Preferred Reporting Items for Systematic Reviews and Meta-Analyses (PRISMA) guidelines (Moher et al., 2009). We assessed patient and provider perspectives of emergency care surrounding IPV to uncover common themes relating to both positive and negative experiences. To evaluate common contributors to positive and negative care experiences, we aimed to be as inclusive as possible in our search strategy regarding social identities, demographic factors, provider types, and diversity of experiences.

### Search Strategy

During September 2021, we searched Embase, PsychInfo, Medline, CINAHL, and Cochrane for peer-reviewed primary studies published between January 2000 and March 2021. Furthermore, snowballing of landmark studies and grey literature searches including conference proceeding searches were completed sequentially through Google Scholar, ProQuest Dissertations, and Theses Global. We conducted exhaustive searches across these databases using a variety of MeSH terms and keywords including but not limited to: *Intimate Partner Violence, attitudes, experiences*, and *Emergency Department*, details of which are found in Appendices A and B.

### Inclusion and Exclusion Criteria

Inclusion criteria comprised any study detailing first-person perspectives, experiences, attitudes, or stories on interactions between individuals identifying as having experienced IPV (current or past) and any professional involved in medical care or paramedical care during or surrounding ED visits for any reason. These providers could include physicians, emergency medical services, nurses, nurse practitioners, social workers, physiotherapists, occupational therapists, emergency medical technicians (EMTs), police workers, or administrative workers. All study designs and all participant demographics were included, without restriction by age, gender, sexual orientation, race, language, or disability status. Studies without available full texts or not in English/French were excluded. We limited inclusion to the year 2000 and later in an attempt to reflect the current socio-cultural context of ED care.

### Assessment of Methodological Quality

Two independent reviewers (ED and AN) completed title/abstract screening and, subsequently, full-text screenings for eligibility using Covidence (Veritas Health Innovation, 2021). Disagreements between reviewers were resolved through team adjudication after independent review. Inter-rater agreement of study inclusion was confirmed following screening.

Quality assessment of included studies was conducted using the Critical Appraisal Skills Program (Critical Appraisal Skills Program, 2021). One reviewer (ED) completed the critical appraisal on all studies with 30% reviewed in duplicate by a second independent reviewer (AN) for quality assurance. Discrepancies were resolved by consensus or by an independent third-party review. Each study’s risk of bias was evaluated as low, medium, or high by combining ethical considerations and critical appraisal findings, as well as specific limitations including lack of saturation, lack of independent recruiters or analysis, and presence of selection or misclassification bias. Studies meeting all 10 CASP criteria were considered highest quality. Due to the small number of studies meeting inclusion criteria, no study was excluded based on lower methodological quality or CASP scores.

## Results

### Study Inclusion

**Figure 1.**
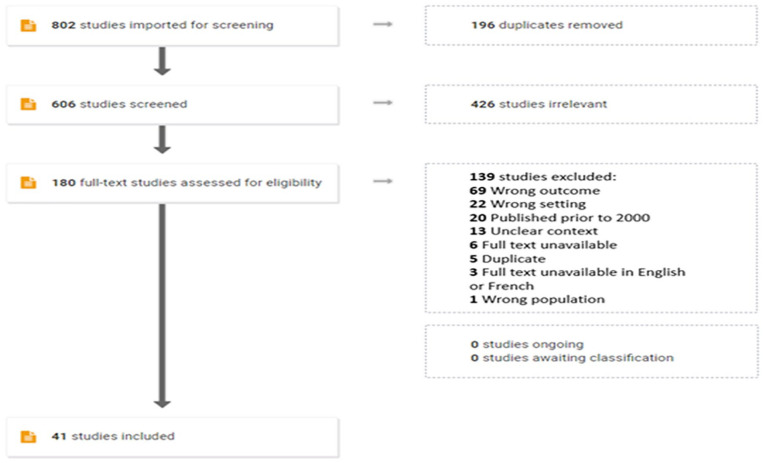
PRISMA diagram. PRISMA = Preferred Reporting Items for Systematic Reviews and Meta-Analyses.

A total of 802 studies were imported into Covidence, including 196 duplicates. In title and abstract screening, 426 studies did not meet study inclusion/exclusion criteria. Full-text screening was completed on 180 studies and yielded 41 final studies meeting all inclusion criteria. Cohen’s Kappa measuring inter-rater reliability for title/abstract and full-text screening were 69% and 65%, respectively. Reasons for exclusion are provided in Figure 1 PRISMA diagram and individual details surrounding included studies are shown in Table 1.

**Table 1.** Included Studies.

Author (Year)	Country	Method of Data Collection	Population	*n*	Bias
**Patient studies**					
Bacchus et al. (2003)	UK	I	Women booking for maternity care hospitals	16	High
Bateman and Whitehead (2004)	UK	I	New mothers registering for health visitor service that disclosed IPV	56	High
Catallo et al. (2012)	Canada	I	English-speaking women aged 18–64 years who screened positive for IPV	19	Med
Catallo et al. (2013)	Canada	I	English-speaking women aged 18–64 years with experiences of IPV disclosure events in urban emergency	19	Med
Du Mont et al. (2013)	Canada	Q	Women aged over 12 presenting to hospital sexual assault and domestic violence treatment centers within 7 days of an assault	920	Med
Du Mont et al. (2014)	Canada	Q	Any client, any age using hospital sexual assault and domestic violence treatment centers	1484	Med
Koziol-McLain et al. (2008)	New Zealand	I	Women presenting to ED and screened positive for IPV and women presenting to Maori healthcare clinic	36	Low
Leppäkoski et al. (2011)	Finland	I, Q	Women aged 18 years and older presenting with acute physical IPV	42	Med
Loke et al. (2012)	China	I	Women aged 18 years and older living with assailant presenting to the ED for physical injuries related to IPV	9	Med
Mayer (2000)	USA	I	Female residents of a shelter for IPV	35	High
O’Doherty et al. (2016)	Australia	I	English-speaking women who screened positive for IPV, not eligible for existing RCT	14	Low
Olive (2017)	UK	I	Women currently using community-based IPV services centers having gone to the ED after an incident of IPV	6	Med
Pratt-Eriksson et al. (2014)	Sweden	I	English-speaking women aged over 18 years living at a women’s shelter having experienced IPV	12	Low
Reisenhofer and Seibold (2013)	Australia	I	Women having sought ED or primary health care while experiencing IPV	7	Med
Roush (2009)	USA	Personal piece	Personal piece of single woman visiting ED after experiencing IPV	1	High
Tower et al. (2006)	Australia	I	Women using non-governmental organization support services for IPV	9	Med
Yam (2000)	USA	I	Women living in shelter having presented to ED for IPV-related injuries at least once in the past year	5	Med
**Provider studies**					
Broissoie & Roberto (2015)	USA	I	Community professionals likely to encounter older women having experienced IPV including medical, legal, and clergy	87	Low
Colombini et al. (2013)	Malaysia	I	Health providers interacting with women experiencing violence including nurses, physicians, social workers, and hospital managers	54	Med
Corbally (2001)	Ireland	Literature review	N/A	N/A	High
D’Avolio (2011)	USA	I, Q, field observations	English-speaking healthcare providers delivering IPV services including patient care, administration, advocacy, and policy	23	Med
Dawson et al. (2019)	Australia	I	Clinical staff working ED (including emergency physicians, nurses, and social workers)	35	Low
Efe and Taşkın (2012)	Turkey	I	Nurses having worked in the ED over 1 year	30	Med
Fay-Hillier (2016)	USA	I	ED nurse aged over 18 having assessed least 500 ED patients in the last year with intake form containing IPV screening question	21	Low
Goldblatt (2009)	Israel	I	Female nurses working with abused women in different healthcare setting	22	Med
Inoue and Armitage (2006)	Japan, Australia	I	Nurses working in accident and EDs	41	High
Leppakoski and Paavilainen (2013)	Finland	Q	ED professionals including nurse, practical nurses, and EMTs	480	Med
Loughlin et al. (2000)	New Zealand	I	ED professionals participating in IPV protocol including staff, project group members, referral service staff, and social worker	18	Med
Maina and Majeke (2008)	Kenya	I	Health professionals having worked in the ED at least 1 year	11	High
Maina (2009)	Kenya	I	ED health professionals	11	Med
McGarry and Nairn (2015)	UK	I	Nursing staff working in a large UK ED including staff grade nurses, specialist or advanced nurse practitioners, and ED assistant	16	Med
McGarry (2017)	UK	I	Health professionals working in acute care within a single NHS Hospital who participated in IPV training sessions	11	Med
O’Malley (2011)	USA	I, Q	Nurses and physicians working at large regional pediatric ED or urgent care center	132	Low
Ritchie et al. (2009)	New Zealand	I	ED permanent nurses and social workers having attended Family Violence Intervention Program training (or scheduled to attend)	11	Low
Robinson (2010)	USA	I	ED nurses	13	Med
Sormanti and Smith (2009)	USA	I	Physician residents in emergency medicine working in urban ED	25	Med
Tower et al. (2012)	Australia	I	Emergency and community health nurses working at urban hospital ED	12	Med
van der Wath et al. (2013)	South Africa	I	ED nurses having cared for IPV survivors in the past year employed at urban district public hospital or tertiary care ED	11	Low
Zijlstra et al. (2017)	Netherlands	I	ED staff in university hospital including physicians, physicians in-training, receptionists, and operational managers	18	Low
**Patient and provider studies**					
Dowd et al. (2002)	USA	I	English- or Spanish-speaking mothers/guardians aged 18–65 years bringing their child to pediatric ED seen by physicians and nurses	59 pts 38 pvs	Low
Rhodes et al. (2007)	USA	Audiotape analysis	Stable English-speaking female patients aged 16–69 years discussing/disclosing IPV in urban academic or community ED	1281 pts 82 pvs	Low

*Note*. I denotes in-person, virtual or telephone interview or focus-group-based data collection; Q denotes questionnaire or survey-based data collection; pts denotes patient participants; pvs denotes provider participants; Med denotes medium bias risk. ED = emergency department; EMTs = emergency medical technicians; IPV = intimate partner violence; RCT = randomized controlled trial.

### Characteristics of Included Studies

Of the 41 included studies, 17 focused on patient experiences, 22 focused on provider experiences, and two included both patient and provider ED care experiences. There were 15 studies completed in North America, 10 in Central and Eastern Europe, nine in Australasia, three in East and South-East Asia, two in the Middle East, and one in Africa. While study designs varied, qualitative methodology was predominant, followed by mixed-methods studies. Personal and focus-group interviews were the most frequent data collection method (34/41, 83%) followed by cross-sectional surveys or questionnaires (7/41, 17%). Other study designs included one publication featuring a personal narrative, one featuring a literature review, and one secondary qualitative analysis of audiotapes between patients and providers from a randomized controlled trial evaluating a health risk assessment tool.

### Patient Study Demographics and Characteristics

While inclusion and exclusion criteria varied, studies on patient experiences almost always recruited women, with only a single study (Du Mont et al., 2014) featuring perspectives from men and/or transgender individuals. Two studies (Koziol-McLain et al., 2008; Leppäkoski et al., 2011) excluded participants based on their inability to speak English or experiencing substance use or mental illness. In all, 13 studies featured data from participants experiencing ongoing IPV at the time of data collection, while six did not explicitly detail whether IPV was ongoing. Seven studies collected information on education and dependents, with most participants having at least secondary education and one or more children.

Commonly collected patient demographics were gender and age, while sexual orientation was not reported in any study. Only two studies (Du Mont et al., 2013, 2014) collected experiences from people identifying as having a disability. Three studies reported the mean ages of participants (30.7, 34 and 30 years) (Catallo et al., 2012; Koziol-McLain et al., 2008; Rhodes et al., 2007) and three reported the median ages of participants (28.5, 39 and 28 years) (Bacchus et al., 2003; Leppäkoski et al., 2011; Pratt-Eriksson et al., 2014). In all, 11 studies collected details on race or ethnicity. Of these, few featured experiences from participants identifying as Black, Indigenous and People of Color (BIPOC).

### Provider Study Demographics and Characteristics

Studies mostly featured the perspectives of nurses (21/22, 95%) and physicians (11/22, 50%) caring for IPV-affected patients. Seven studies collected data solely from nurses (Efe & Taşkın, 2012; Fay-Hillier, 2016; Goldblatt, 2009; Inoue & Armitage, 2006; Robinson, 2010; Tower et al., 2012; van der Wath et al., 2013). Social workers were included in three studies (Colombini et al., 2013; D’Avolio, 2011; Dawson et al., 2019); EMTs in two studies (Broissoie & Roberto, 2015; Leppäkoski & Paavilainen, 2013); administrative professionals in three studies; spiritual care professionals in two studies (Broissoie & Roberto, 2015; McGarry, 2017); and justice system professionals in one study (Broissoie & Roberto, 2015).

Gender was not collected in approximately one-third of the studies. Among those that reported gender, male provider perspectives were often missing; 10 studies only successfully recruited female providers and two directly excluded male participants (Goldblatt, 2009; Inoue & Armitage, 2006). No studies featured trans or gender diverse providers. Only one study collected sexual orientation demographics, where 1/21 participants identified as gay (Fay-Hillier, 2016).

Provider race or ethnicity was explicitly collected in four studies (D’Avolio, 2011; Fay-Hillier, 2016; Rhodes et al., 2007; van der Wath et al., 2013). Seventy-five percent of these mainly featured participants identifying as European, Caucasian, or white. No provider study collected information regarding disability.

### Methodological Quality of Included Studies

All 10 criteria of the CASP checklist were met in eight studies: one patient-centered study (Koziol-McLain et al., 2008), five provider-centered studies (Dawson et al., 2019; Fay-Hillier, 2016; O’Malley, 2011; Ritchie et al., 2009; Zijlstra et al., 2017), and both of the patient–provider studies (Dowd et al., 2002; Rhodes et al., 2007).

Validated or piloted data collection tools were used in 11 studies: four patient-centered studies (Du Mont et al., 2013, 2014; Loke et al., 2012; Olive, 2017), six provider-centered studies (Broissoie & Roberto, 2015; Colombini et al., 2013; Efe & Taşkın, 2012; Leppäkoski and Paavilainen, 2013; O’Malley, 2011), and one patient–provider study (Dowd et al., 2002).

In all, 14 studies reached saturation: five patient-centered studies (Bacchus et al., 2013; Catallo et al., 2012, 2013; Reisenhofer & Seibold, 2013; Yam, 2000) and nine provider-centered studies (D’Avolio, 2011; Dawson et al., 2019; Fay-Hillier, 2016; Goldblatt, 2009; Inoue & Armitage, 2006; Loughlin et al., 2000; Ritchie et al., 2009; van der Wath et al., 2013; Zijlstra et al., 2017).

In total, 14 studies used independent analysts: five patient-focused studies (Catallo et al., 2012, 2013; Koziol-McLain et al., 2008; Loke et al., 2012; Pratt-Eriksson et al., 2014), seven provider-focused studies (Broissoie & Roberto, 2015; Goldblatt, 2009; Loughlin et al., 2000; O’Malley, 2011; Ritchie et al., 2009; Sormanti & Smith, 2009; Zijlstra et al., 2017), and both patient–provider studies (Dowd et al., 2002; Rhodes et al., 2007).

### Findings of the Review—Patient Perspectives

Contributors to positive and negative care experiences were identified at the individual, institutional, and systemic levels. Sample quotes and principal findings are provided in Table 2.

**Table 2.** Critical Findings—Patient and Provider Care Experiences.

Level	Domain	Recurring Experiences	Selected Sample Quote	Reference
**Positive patient experiences**				
Individual	Patient-centered, TIC	Pvs perceived as supportive and non-judgmental	“For a horribly humiliating experience your staff did a great job in comforting, [and] explaining things. Not judging and certainly not forcing any procedure on you that you were not willing to do. I thank you for being there through a terrible time”	Du Mont et al. (2014)
		Pvs offer opportunities to discuss IPV openly	“I did feel safe and believe it or not it felt good to finally release it. I’ve never been able to express it before. I’ve told a few ones about having been abused, but not going into the details and all that”	Koziol-McLain et al. (2008)
		Pvs experienced as empathetic, caring, and seeing person as a whole	“One thing I will say that stood out for me in that hospital was that particular doctor, he was just really, really nice. He came in, he was friendly, he asked me how I was, which was important because nobody was really asking me how I was, you know what I mean”	Olive (2017)
		Pvs validating patient are experiences, and that it consists of abuse	“He [ED nurse] told me how anything that is abusive is not acceptable and I got all teary and started to understand that maybe I was a victim of domestic violence”	Reisenhofer and Seibold (2013)
Institutional	ED infrastructure and practices	Pts having access to private space	“I was given a peaceful room where to be alone”	Leppäkoski et al. (2011)
		Pts do not have to face long wait times	“I received care very quickly”	Leppäkoski et al. (2011)
	Access to effective and timely specialized care	Knowledgeable pvs offering useful and accessible resources in timely manner	“[T]he nurse who saw me [. . .] was truly an angel. She was so extremely kind and professional. She spoke to me about everything and more. I was upset, and she made me feel so safe, so happy and so cared for. She had so much information that helped me understand what had happened to me[. . .]”	Du Mont et al. (2013)
		Access to thorough medical care	“Thoroughness, bruises were examined carefully”	Leppäkoski et al. (2011)
Systemic	Accessible and timely specialized care	Efficient and effective referral and follow-up systems	“One woman suggested that a follow-up call is needed; [. . .] some way to follow up, like a few days later call me and ask me how I feel and what I want to do”	Yam (2000)
**Negative patient experiences**				
Individual	Lack of patient-centered, TIC	Pvs perceived as uncaring or unfriendly	“When the physician came she was so cold.[. . .]”	Pratt-Eriksson et al. (2014)
		Pts experiencing pvs as controlling	“They can’t be pushy on what you tell somebody to do—they did that a lot”	Yam (2000)
		Pts feel pressured by pvs to lay charges	“When they found out that I didn’t want to press charges, they started treating me like trash. I was sore all over. I couldn’t really move and I kept telling them that I wanted something for pain and I could hear them talking over there. The doctors and nurses, they were like, “If she doesn’t want to press charges, she doesn’t need pain medication”	Mayer (2000)
	Provider lack of knowledge surrounding IPV	Pvs perceived as judgmental	“I had the nurse tell me I was stupid to be in this situation because I was pregnant”	Mayer (2000)
		Pvs perceived as offering unrealistic advice	“[. . .] [Providers] say stupid things like 'leave him, you deserve better'. Ha! What do they know”	Bateman and Whitehead (2004)
		Pts experiencing pvs as not respecting their autonomy or being patronizing	“I had stitches in my head. While the doctors were sewing me up they were talking over me like I wasn’t even there saying “I don’t understand why these women put up with this, why don’t they just leave them [the perpetrators]? They were mean. I was awake, not unconscious. It wasn’t like I couldn’t hear anything. They talked about me as if I was dead”	Mayer (2000)
		Pvs perceived as ignoring IPV as the underlying cause for ED presentation	“I told the nurse what happened. I told her my son’s father beat me up. He had pushed me on the cement. I sprained my knee. She just wrote down what was hurting me and my vital signs and that was it. She didn’t ask any more questions”	Yam (2000)
	Bias and prejudice experienced on individual level	Pvs perceived as blaming pts	“[. . .] I feel like a lot of the hospital staff feel, like why would you stay? It’s your fault, you know? One nurse hollered at me and said: “Are you crazy?” “What’s the matter with you?” [. . .]”	Yam (2000)
		Pvs perceived as dismissive or not believing pts	“I had a burn once and I had some of the gauze melted into the skin and they tried to take it off and when I kept telling them that it hurt, they were like, “no it doesn’t” and I’m like, “yes it does”; they were like “no it doesn’t.” I found that kind of thing was quite regular [. . .] So I kind of felt a little ignored” “The doctor made me feel like I was a drug seeker”	O’Doherty et al. (2016)
		Pvs perceived as not respecting autonomy or being patronizing toward pts	“I had stitches in my head. While the doctors were sewing me up they were talking over me like I wasn’t even there saying “I don’t understand why these women put up with this, why don’t they just leave them [the perpetrators]? They were mean. I was awake, not unconscious. It wasn’t like I couldn’t hear anything. They talked about me as if I was dead”	Mayer (2000)
Institutional	Time	Long wait times	“I arrived after 12 A.M. and we didn’t fully get looked after for 9 hours, so most of the evidence was dried up”	Du Mont et al. (2014)
		Pvs perceived as impatient or rushed	“[. . .]It seems as if they want to get you out”	Yam (2000)
	Infrastructural	Lack of privacy	“Every single patient around the waiting room must have heard the doctor ask if my husband had punched me”	Leppäkoski et al. (2011)
		Lack of safe interviewing policies	“He [the perpetrator] came in to see me and he started arguing with me and hit me. When the nurse came in, I couldn’t say anything. He would have hit me again and probably the nurse too.[. . .]”	Mayer (2000)
	Lack of education and policies surround IPV, with pvs perpetuating bias care	Pvs uncomfortable discussing, not recognizing or ignoring IPV	Patient: Hit me in the face . . . that was like . . . almost a year ago. Provider: All right. But that’s not really domestic. Patient: Oh. Provider: Right? Patient: No. Right— Right— N-Not— I don’t—. Provider: Okay, so that’s (. . .). Okay. (1-second pause) Any coughing or shortness of breath?	Rhodes et al. (2007)
		Pvs minimizing gravity of IPV	*The provider asked during the examination*: “Any problems at all with domestic violence? I have to give him the evil eye when I ask that question.” The patient laughed, and the provider then addressed her male partner and asked. “Now, is she givin’ you any trouble?” He responded, “Yep”	Rhodes et al. (2007)
Systemic	System not properly addressing the needs of patients	Lack of accessible, effective, timely, interdisciplinary 24/7 specialized care	“It took two hours for the [SA/DV] staff to come to hospital.. . . Hospital [emergency department] nursing staff called all numbers available for the sexual assault team, no one picked up”	Du Mont et al. (2014)
		Pts perceiving services as useful for their needs	“The social workers just told me to escape from violent scenes. This is impossible, for he can grab me. Another told me to get a divorce. This means they can’t help”	Loke et al. (2012)
		Pts feeling failed or betrayed by the system	“I didn’t do anything about the violence because the police here are kind of funny. They are tired of domestic violence. If you call the police for domestic violence, they take you both to jail. Period. So the only thing I could do was sneak out. [. . .]”	Mayer (2000)
	Societal and cultural norms and perpetuation of stereotypes and myths leading to repeated bias and discrimination within healthcare and legal systems	Repeated invalidation of pts experiences due to their gender and failure to take action due to patriarchal societal norms	*Several women also experienced the impact of discourses that viewed IPV as being incited by behavior of “crazy” or mentally ill women, which reinforced their belief that they needed fixing rather than care.* “He (ED doctor) never acknowledged I’d been abused. He just checked the physical side of things [. . .] he asked me if I was taking my sleeping tablet [. . .] it was all my fault because I wasn’t sleeping, I was a tired and grumpy woman, there was nothing wrong with [assailant].” “I called the police for help, but they only told us to stop quarreling. I begged them to help, but all they said was, ‘Just forgive him, all men are like him; being a woman, you should know’. The police were, like, forming an alliance with him”	Reisenhofer and Seibold (2013) Loke et al. (2012)
**Positive provider experiences**				
Individual	Having good knowledge and comfort levels surrounding IPV	Feeling well-equipped to discuss IPV	“[Some] staff are certainly not interested [in asking]. I think a lot of them can’t handle it themselves [. . .] and I think you’ve got to feel comfortable with yourself in order to ask those questions. [. . .] I feel quite comfortable questioning, and yet a few years ago perhaps I wouldn’t have. I think a lot of that just comes from life’s experiences”	Loughlin et al. (2000)
		Supporting patients to find resources they need	“I think it’s essential that we screen, [. . .] but our role is to help them find the appropriate resources, whether that be patient social work, calling family members for them, contacting the police if it’s a rape case, even if it’s a regular assault case. So [I am] more of an interventionist in the sense of trying to put them down the right path to get the resources that they need.[. . .]”	Fay-Hillier (2016)
		Understanding the complexity of IPV	“In a few cases, I’ve been able to get them resources that were probably appropriate for them. [But] quite frankly, a lot of times people, if they do say yes, and you offer to call police and things like that or [have them] go to a shelter, they say no. People don’t want to go to a shelter. A lot of people would rather go home to their abusive partner than go to a shelter. . . . They hear the word shelter and they—maybe there’s a different word we could start using. But shelter, they think about like homeless shelters and they think, “That’s not me. I’m not poor.” Or there’s kids involved, which makes it even more complicated, because now maybe you have a married couple with kids and no one person has custody. So if the abused partner leaves, he or she can’t just take the kids along unless the kids are being abused”	Fay-Hillier (2016)
	Offering care leading to positive patient experiences	Helping pts feel comfortable to discuss IPV openly	“She (the patient) was relieved. She knew I knew and she just wanted to tell somebody. She wanted somebody to know what was happening”	Loughlin et al. (2000)
		Feeling that they can make a difference	“[. . .] If I can show one woman that she can get out and have a good and happy life, then that’s fine [. . .]”	Loughlin et al. (2000)
		Having the ability to improve patient safety, comfort, and well-being in the ED	“. . . just making them feel safe and [. . .] helping them if they needed bandages. [. . .] you know giving them clean things to put on or saying do you need a shower, can I get you something to eat or drink. [. . .] Yeah making them feel comfortable”	Tower et al. (2012)
Institutional	Accessible, effective and timely referral and follow-up systems for high-quality services surrounding physical health, mental health, and legal proceedings	Efficient referral systems allowing best patient care while preserving ED resources	“People from Victim’s Support or Rape Crisis, when they come in, they can do all the little bits and pieces [. . .] provide them with transport, provide them with the bed for the night, provide them with the support, provide them with the understanding, and I think that’s what those people need in that initial stage. That is something we as nursing staff don’t have the time and facilities and ability to be able to offer them on any great scale”	Loughlin et al. (2000)
		Better infrastructure to offer safe, private spaces	*The newly installed private consulting rooms at the ED were named by most participants as facilitating, as private rooms created a safer environment than beds separated by a curtain only*	Zijlstra et al. (2017)
		Policies, resources and educational opportunities available on institutional level	*The resources available within the ED were considered to be helpful, with information readily available as a resource for people experiencing abuse. Other resources cited include cue cards, senior staff support, reminder posters around the department, and information about community resources that women could use.* “For years I would be afraid to ask, even if I suspected him and I would never discuss it with the woman. [. . .] I don’t know why but now I do. [. . .] [Is it the protocol that has brought about that change?] Yes I think so, definitely”	Ritchie et al. (2009) Loughlin et al. (2000)
Systemic	Accessible, effective, timely, interdisciplinary 24/7 specialized IPV care	Collaborating with different providers including specialized IPV-care teams	*There was a strong sense of collaboration between staff in the ED in both hospitals when describing the response to IPV* “the team part, that communication between us all is really important” “I think social work is our biggest fallback and our biggest support”	Dawson et al. (2019)
		Accessible shelters and community services Efficient follow-up and referral structures	*Pediatric emergency providers valued* “linking at risk families to community resources”	O’Malley (2011)
**Negative provider experiences**				
Individual	Coping with negative emotions	Feeling powerless, frustrated, intimidated, or apathetic surrounding IPV care, including lack of understanding regarding pts declining police/shelters returning to violence, or having repeated ED visits for injuries	“It is our normal practice here [. . .] in reality, it sounds terrible to say but you are almost feeling like you’re probably wasting your breath [. . .] talking to them.” “There is a known recidivist with proven family violence who comes to our ED twice a week. We offered a lot to her, but she keeps coming back and I think: “Oh, it’s her again.” Now I’ve given up [. . .] and stopped paying attention to the violence	Inoue and Armitage (2006) Zijlstra et al. (2017)
		Fearing liability	“I want to know exactly what’s expected of me in terms of the assessment and the related documentation and I want to know who I can turn to if and when I need assistance. I don’t want to be asked to do something that hasn’t been thought through carefully especially when it could result in me being liable for a woman’s life as well as my own career”	Sormanti and Smith (2009)
		Feeling uncertain about their ability in ensuring safety or intervention	“[Suppose that somebody comes in] and they say, ‘Well, no, I don’t feel safe.’ So we just asked them that question; they just opened up to us. Well, now it’s up to us to help them [. . .] But what if we can’t help them? Then we’re just going to send them back out in the streets and they’re going to be like, ‘Okay, I just opened up but they can’t help me, so I guess I’ll just have to go back to my house”	Fay-Hillier (2016)
	Navigating intersecting social and professional identities	Experiencing intrusive memories and strong emotions from traumatizing cases	“ [. . .] the injuries that I have seen [. . .] The moment you are alone it comes back[. . .]” “I will never forget it. . .It looked awful. Of all my encounters with abused women, this was the most devastating because her face was destroyed. [. . .] I swear that for two whole days I couldn’t look at my face in the mirror. It kept turning into the abused woman’s face, full of blood [. . .] This passed eventually”	van der Wath (2013) Goldblatt (2009)
		Having difficult to separating between work and home	“I am trying very hard to separate work from family life. I am a mother and I do my best not to blur the boundaries in all my work domains. [. . .] It’s very difficult for me. And when I return home from work I can hug and kiss my kids and thank my lucky stars [. . .]”	Goldblatt (2009)
		Feeling many emotions causing some to develop impersonal approach	“I like not to get emotionally involved, because you see so many people and you come across so many potentially distressing situations that the more you can emotionally shut out the better”	Tower et al. (2012)
	Bias and discrimination displayed on individual level	Pvs stereotyping patients experiencing IPV	“[. . .] all the victims were kind of similar [. . .] [they] come in by ambulance, swearing, drunk, bruised [. . .] they all looked the same [. . .] all very skinny, they were all lower socio economic area type people, they swore a lot[. . .] all had used drugs or do use drugs, or they all drank alcohol, or [. . .] I hate to say the words and I am sorry to say the words but they fit that whole white trash picture [. . .] it’s a stereotype but you know my brain has worked along those lines [. . .]”	Inoue and Armitage (2006)
		Pvs feeling that pts experiencing IPV lack credibility or honesty	“[. . .] some people really want attention. [. . .] when you ask the same questions to everyone, sometimes it just offers an invitation for more attention-seeking behaviors”	Fay-Hillier (2016)
		Pvs blaming pts or believing them to be at fault	“I don’t have time to hear a 30-minute story about it. You’re a grown person: get out of it. That’s horrible, I shouldn’t be saying that”	Fay-Hillier (2016)
		Cultural belief that IPV disclosure harms family structures or is intrusive	“I think it’s still a big taboo for many people. The general opinion is: You shouldn’t interfere; it’s none of our business”	Zijlstra et al. (2017)
Institutional	Competing demands: time and acuity	Having little time	“The other issue is those nurses and doctors are worked to death. They are really, really busy; they are out straight. They have very little time to deal with anything. Then that’s the other issue, we have to look at that.” “[. . .] I don’t have the time to hold your hand or wait for social services to come to see you if there are tons of other people waiting”	D’Avolio (2011) Sormanti and Smith (2009)
		Feeling overwhelmed from routine responsibilities, IPV care seen as burden	“say you ask (about IPV) and they say yes? I can’t run out the door, you know. I have to stop, and then I’m stuck with them	Sormanti and (2009)
	Lack of resources	Lack of policies, ED-based services and referral systems to appropriately manage IPV	“There was no protocol, no overview of agencies, and no established cooperation with social welfare agencies. This made the ED employees feel incapacitated in taking the right steps in managing IPV. All people here want to mean something, do something. But because we lack materials, background, or information, possibilities are few. That’s why IPV takes the backseat.”	Zijlstra et al. (2017)
		Lack of training, education, knowledge, and comfort regarding IPV	“I sometimes feel I don't know what I can do to help this person. It's like I am not sure if I am helping her enough or I am not sure whether I am adequately prepared. I would like to have training and know what I am supposed to do”	Maina (2009)
		Lack of infrastructure and resources to offer safe, private care	*They were unable to be left alone with the women even if they wanted to intervene as there was no suitable environment and they were therefore afraid that the woman’s relatives could hurt them or the woman.* I haven’t got the time and I haven’t got the facilities—the private room, the good surroundings, the safe environment.”	Efe and Taşkın (2012) Loughlin et al. (2000)
Systemic	Navigating inefficient system	Inefficient referral systems, inadequate services and poor follow-up care	“[. . .] to do a social service referral [for domestic abuse], you dread it because of the length of time it takes, the telephone calls that it then takes, the messages left on answering machines, waiting for someone to call you back, blah, blah, blah”	McGarry and Nairn (2015)
		Challenges with legal system	“[. . .] when you report partner violence to the police it is not taken seriously and even at the police station there is nobody to counsel the couple”	Maina (2009)
	Longstanding under-funded EDs leading to poor infrastructure and resource availability	Chronic lack time, privacy, space, or funding for specialized IPV care in the ED	“Hospital policy and IPV training protocols were followed, only to learn from the social service department that there was no longer a social worker to cover the unit because of budget cuts. So here we are in X unit at X hospital and so we do our screening and the scary part was to learn the social worker all of a sudden got pulled from our department, budget cut. [. . .] So I’ve asked this great question about violence, and now I don’t have any services. There is this young victim who has told me and is at such high risk. It’s very frightening to think that I don’t know what will happen to her. It’s kind of scary.” *Organizational productivity expectations were constantly revised upward in both settings, which resulted in expectations to provide more care, to more patients, in less time*	D’Avolio (2011)
		Lack of appropriate staffing in the ED	*Nurses focused on the high volume of patients needing to be screened and their level of acuity as barriers*: “[. . .] in triage, it’s really—it’s just hard, when you have 50 in the waiting room and it’s just—sometimes there’s supposed to be two nurses out there, but sometimes if we’re short-staffed or I have some things going on, sometimes you just can’t”	Fay-Hillier (2016)
	Societal and cultural norms and perpetuation of stereotypes and myths leading to repeated bias and discrimination within healthcare and legal systems	Repeated invalidation of pts experiences due to their gender and failure to take action due to patriarchal societal norms	*Only a quarter of the providers [. . .] mentioned sexual abuse among the types of acts that may characterise domestic violence. One medical respondent clearly stated that rape was not a form of family violence.* “I: Would it also be sexual? Would also be considered? R: Sexual abuse, rape, I don’t think, sexual abuse no”	Colombini et al. (2013)

*Note.* pts denotes patients; pvs denotes providers. EDs = emergency departments; IPV = intimate partner violence; TIC = trauma-informed care.

### Positive Experiences

In general, patients perceived opportunities for open discussions on IPV as positive (Koziol-McLain et al., 2008) and long overdue (Koziol-McLain et al., 2008). Two studies in this review described the psychological processes behind the decision to seek care and establishing readiness to discuss IPV or to leave a violent relationship. Using the Transtheoretical Model of Change, the first study (Catallo et al., 2012) found that individuals often disclose IPV after weighing the benefits against the risks of disclosing, the latter including examples such as fear for their safety, fear of child apprehension, and/or fear of judgment from healthcare providers. The second study (Catallo et al., 2013) described disclosure and care seeking as a four-phase process, with minimizing the risk of intrusion by healthcare professionals and evaluating the level of trust with these professionals at the center. Patients felt that disclosing violence to an ED provider could cause intrusion, defined as involvement of healthcare professionals resulting in further chaos and trauma. Risk of intrusion was weighed against the ability of the provider to offer services or interventions perceived as useful and positive. In a third study, healthcare interactions were found to have important effects on individual identity, helping victims acknowledge IPV and begin to re-construct their sense of self (O’Doherty et al., 2016).

On an individual level, patients commonly described interactions with non-judgmental, empathetic and caring providers as contributors to positive ED experiences (Catallo et al., 2012; Leppäkoski et al., 2011; Reisenhofer & Seibold, 2013; Olive, 2017). Patients appreciated having a provider validate their experience as consisting of IPV and pausing in their busy shift to acknowledge IPV as wrong or unjustified (Bacchus et al., 2003; Koziol-McLain et al., 2008; Reisenhofer & Seibold, 2013; Yam, 2000). Many patients perceived experiences positively when their providers cared for them holistically, seeing them as a whole person and not just as their injuries. This included having access to objective and thorough medical care (Leppäkoski et al., 2011) and comprehensive psychological and social support to meet their needs (Du Mont et al., 2013; Koziol-McLain et al., 2008; Mayer, 2000; Olive, 2017; Tower et al., 2006; Yam, 2000).

On institutional and systemic levels, many patients positively described experiences with providers who were knowledgeable on IPV (Du Mont et al., 2013; Leppäkoski et al., 2011). This included positive interactions with dedicated and kind law enforcement workers offering support before, during, and after ED care (Mayer, 2000; Olive, 2017). It was important for patients to have the ability to be seen quickly (Leppäkoski et al., 2011), and to have providers who took the time to listen to them (Olive, 2017). Patients also appreciated access to private spaces helping them feel safe (Leppäkoski et al., 2011). Readily available local resources (Du Mont et al., 2013; Leppäkoski et al., 2011), access to timely specialized IPV care around-the-clock (Leppäkoski et al., 2011), and an option for close follow-up with timely community referrals (Yam, 2000) all played significant roles in ensuring positive care experiences.

### Negative Experiences

A concerning number of negative patient experiences were uncovered, commonly arising from perceived adverse provider behavior, as well as institutional and system-level issues encountered when navigating the health system.

On an individual level, negative provider behaviors commonly perceived by patients included providers minimizing violence (Leppäkoski et al., 2011; Rhodes et al., 2007; Yam, 2000), appearing unconcerned (Mayer, 2000; Reisenhofer & Seibold, 2013; Yam, 2000), displaying blame (Bateman & Whitehead, 2004; Leppäkoski et al., 2011; Mayer, 2000; Reisenhofer & Seibold, 2013; Yam, 2000), expressing pity (Du Mont et al., 2013; Mayer, 2000), or exhibiting judgment (Du Mont et al., 2013; Mayer, 2000; Pratt-Eriksson et al., 2014; Reisenhofer & Seibold, 2013; Rhodes et al., 2007; Roush, 2009; Tower et al., 2006; Yam, 2000). Patients also cited having negative experiences when providers appeared cold (Mayer, 2000; Pratt-Eriksson et al., 2014; Reisenhofer & Seibold, 2013) or lacked compassion (Pratt-Eriksson et al., 2014; Yam, 2000).

Patients also commented on providers seeming uncomfortable or lacking knowledge, education, and experience with the subject of IPV (Bateman & Whitehead, 2004; Mayer, 2000; Pratt-Eriksson et al., 2014; Rhodes et al., 2007; Yam, 2000), with many providers only focusing on physical injuries (Leppäkoski et al., 2011; Loke et al., 2012; Reisenhofer & Seibold, 2013; Yam, 2000). Patients found it particularly harmful when providers ignored violence or failed to address it as the reason for their ED visit (O’Doherty et al., 2016; Leppäkoski et al., 2011; Mayer, 2000; Yam, 2000). While these provider behaviors were mostly recorded from nurses and physicians, one study also outlined EMS as exhibiting similar behavior (Bateman & Whitehead, 2004). Patients also described providers displaying controlling or “pushy” behavior (O’Doherty et al., 2016; Olive, 2017; Pratt-Eriksson et al., 2014; Yam, 2000), with some providers becoming frustrated or uncaring when patients declined police involvement (Mayer, 2000l; Tower et al., 2006; Yam, 2000).

On the institutional level, lack of privacy was a predominant problem, including instances such as sitting in a busy waiting room and perceiving stares from others seeing their injuries, or having to recount violence knowing others could overhear the conversation (Bacchus et al., 2003; Du Mont et al., 2013, 2014; Leppäkoski et al., 2011; Olive, 2017; Yam, 2000). This was also reflected in providers often failing to separate abusive partners from patients during medical examinations (Bacchus et al., 2003; Mayer, 2000; Rhodes et al., 2007; Yam, 2000). Long wait times commonly led to negative experiences (Du Mont et al., 2013, 2014; Leppäkoski et al., 2011; Mayer, 2000), as did interacting with providers who appeared rushed (Du Mont et al., 2014; Mayer, 2000; Yam, 2000). Patients described feeling burdensome and being treated as less important than other patients (Reisenhofer & Seibold, 2013; Tower et al., 2006; Yam, 2000).

On the systemic level, patients reported a lack of effective inter-agency communication and the absence of referral systems for follow-up (D’Avolio, 2011; Du Mont et al., 2014; Leppäkoski et al., 2011; Olive, 2017). Interacting with professionals or services that could not meet their needs further contributed to negative experiences, often leading to feelings of hopelessness (Leppäkoski et al., 2011; Loke et al., 2012; Mayer, 2000; Olive, 2017; Pratt-Eriksson et al., 2014). Negative experiences discouraged individuals from seeking future care (Loke et al., 2012; Tower et al., 2006), with some even resolving to never return to a hospital again (O’Doherty et al., 2016).

Finally, patients described feeling failed by the system (Loke et al., 2012; Mayer, 2000; Olive, 2017) within both the healthcare and justice systems (Catallo et al., 2012; Leppäkoski et al., 2011; Loke et al., 2012; Pratt-Eriksson et al., 2014; Mayer, 2000). For example, some patients felt that both police and ED providers belittled their feelings (Leppäkoski et al., 2011; Loke et al., 2012; Mayer, 2000; Pratt-Eriksson et al., 2014; Reisenhofer & Seibold, 2013), normalized violence (Loke et al., 2012; Rhodes et al., 2007), or vindicated the abuser (Reisenhofer & Seibold, 2013). Patients also experienced stereotyping during ED care, for instance commonly feeling labeled as mentally ill or unintelligent by providers (Mayer, 2000; O’Doherty et al., 2016; Reisenhofer & Seibold, 2013; Yam, 2000). These adverse interactions were compounded, leading to a sense of futility (Leppäkoski et al., 2011; Loke et al., 2012; Mayer, 2000), as well as fear of police involvement within ED care (Catallo et al., 2012, 2013; Mayer, 2000).

### Findings of the Review: Provider Perspectives

We identified contributors to both positive and negative care experiences at the individual, institutional, and systemic levels. Sample quotes and principal findings are shown in Table 2.

### Positive Experiences

On the individual level, nurses and physicians noted positive experiences when able to support patients’ autonomy and offer useful services to patients (Fay-Hillier, 2016; Loughlin et al., 2000; Ritchie et al., 2009; Tower et al., 2012). Providers felt that making a difference even for one person (Loughlin et al., 2000; Ritchie et al., 2009; Tower et al., 2012) and helping to make people feel safe were important to them (Tower et al., 2012). Additional contributors to positive experiences included feeling knowledgeable and well-equipped to manage IPV in the ED (Loughlin et al., 2000; McGarry & Nairn, 2015).

On the institutional level, this was often achieved through education and practice increasing providers’ comfort in asking about and managing IPV (Loughlin et al., 2000; Maina, 2009; McGarry, 2017; Ritchie et al., 2009). Nurses and physicians also described policies and protocols as crucial contributors to positive experiences helping them provide effective and high-quality care (Loughlin et al., 2000; Ritchie et al., 2009). Providers described having adequate time and easy assessment tools (O’Malley, 2011), as well as cooperation (Broissoie & Roberto, 2015; Dawson et al., 2019; Fay-Hillier, 2016; O’Malley, 2011) as key contributors, including ED clinicians placing value on social workers within the interdisciplinary team (Dawson et al., 2019). Positive experiences also transpired when medical personnel, clerical staff, and security personnel successfully collaborated to keep patients safe (Dawson et al., 2019; Tower et al., 2012). Providers also cited having access to on-site, around-the-clock specialized IPV care as contributing to positive experiences (Dawson et al., 2019; Maina, 2009; McGarry & Nairn, 2015; O’Malley, 2011) as did the ability to arrange referrals to community resources (O’Malley, 2011).

### Negative Experiences

On the individual level, providers described asking patients about IPV as opening “pandora’s box” (Dowd et al., 2002; Loughlin et al., 2000; Sormanti & Smith, 2009), “a can of worms” (Sormanti & Smith, 2009), and “a cesspool unable to be solved in the ED” (Zijlstra et al., 2017). Some physicians and nurses believed that IPV was a private, family affair (Efe & Taşkın 2012; Inoue & Armitage, 2006; Sormanti & Smith, 2009; Tower et al., 2012) and viewed it as a social problem rather than a health concern (Robinson, 2010). These providers sometimes held the opined that managing IPV was not their job (Colombini et al., 2013; Dawson et al., 2019; Efe & Taşkın, 2012; Inoue & Armitage, 2006; Sormanti & Smith, 2009; Tower et al., 2012). These attitudes were described by many physicians and nurses as a deterrent to discussing IPV, with many delegating this task to social workers (Dawson et al., 2019; Inoue & Armitage 2006; Maina, 2009).

Nurses, physicians, and social workers noted feelings of frustration and powerlessness when patients declined police involvement (Fay-Hillier, 2016; Robinson, 2010; Zijlstra et al. 2017) or returned to a violent situation (Colombini et al, 2013; Dawson et al., 2019; Fay-Hillier, 2016; Inoue & Armitage, 2006; O’Malley, 2011; Robinson, 2010; Sormanti & Smith, 2009).

Difficulty reconciliating personal experiences with IPV was another individual factor leading to negative provider experiences (Dawson et al., 2019; Leppäkoski & Paavilainen, 2013). Four provider studies collected information on personal history of IPV (Dawson et al., 2019; Fay-Hillier, 2016; Leppäkoski & Paavilainen, 2013; O’Malley, 2011). These found that at least one-fifth of nurses, practical nurses and EMTs had experienced IPV themselves (Leppäkoski & Paavilainen, 2013) and that 21% of male and 27% of female nurses and physicians had personally experienced family violence. Furthermore, over 60% knew someone who had experienced family violence (O’Malley, 2011).

On the institutional level, there was a lack of training and education on IPV which was mentioned in many studies examining experiences of nurses, practical nurses, EMTs, residents, and staff physicians (Dowd et al., 2002; Fay-Hillier, 2016; Inoue & Armitage, 2006; Leppäkoski & Paavilainen, 2013; Loughlin et al., 2000; Maina, 2009; Ritchie et al., 2009; Sormanti & Smith, 2009; Zijlstra et al., 2017). This was reflected in negative physician and nursing provider experiences stemming from lack of knowledge and comfort surrounding IPV (Colombini et al, 2013; Dawson et al., 2019; Dowd et al., 2002; Loughlin et al., 2000; Maina, 2009; McGarry, 2017; Rhodes et al., 2007; Robinson, 2010; Roush, 2009; Sormanti & Smith, 2009; Zijlstra et al., 2017).

Additionally, a lack of guidelines, policies, and infrastructural support often led to negative experiences for nurses and physicians (D’Avolio, 2011; Dowd et al., 2002; Loughlin et al., 2000; Maina, 2009; Zijlstra et al., 2017), as did insufficient standardized protocols for EMT providers. (Broissoie & Roberto, 2015) Physicians, nurses, social workers, and clinical officers cited lack of awareness of existing resources and protocols as negative contributors (Dawson et al., 2019; Loughlin et al., 2000; Maina, 2009). While inter-agency collaboration was usually described as positive, one detective mentioned that the competing missions of law, health, and advocacy professionals could create tension (Broissoie & Roberto, 2015).

Lack of on-site, around-the-clock IPV-care specialists (D’Avolio, 2011; Loughlin et al., 2000; Maina, 2009; Sormanti & Smith, 2009) was particularly frustrating for ED providers, as was the absence of reliable follow-up through effective and timely referral systems (Dowd et al., 2002; Loughlin et al., 2000; Maina, 2009). In this context, physician and nurses frequently invested many hours to find appropriate services for their patients (McGarry & Nairn, 2015; Robinson, 2010; Zijlstra et al., 2017), sometimes remarking that no good local resources existed (D’Avolio, 2011; Dawson et al., 2019; Efe & Taşkın, 2012; Fay-Hillier, 2016; Loughlin et al., 2000; Sormanti & Smith, 2009). Some nurses, doctors, and clinical officers also described dissatisfaction when seeking assistance from police and legal professionals, only for their patients to not be taken seriously or to be declined legal counsel (Maina & Majeke, 2008). These issues compounded intense feelings of frustration and powerlessness in care delivery (Efe & Taşkın, 2012; Fay-Hillier, 2016; Maina, 2009; Robinson, 2010; Sormanti & Smith, 2009; Zijlstra et al., 2017).

On the systemic level, providers described working in chronically under-resourced environments, with significant time constraints as a major barrier to positive experiences (Colombini et al., 2013; D’Avolio, 2011; Dawson et al., 2019; Dowd et al., 2002; Efe & Taşkın, 2012; Fay-Hillier, 2016; Loughlin et al., 2000; McGarry & Nairn, 2015; Ritchie et al., 2009; Robinson, 2010; Sormanti & Smith, 2009; Zijlstra et al., 2017). While this was mostly felt by nurses and physicians, administrative personnel also reported similar experiences (D’Avolio, 2011; Zijlstra et al., 2017). Nurses and physicians also noted they were forced to prioritize unstable patients, challenging their ability to holistically manage IPV (Fay-Hillier, 2016; Ritchie et al., 2009). Limited ED infrastructure with a lack of private spaces also contributed to negative experiences (Efe & Taşkın, 2012; Loughlin et al., 2000; Zijlstra et al., 2017).

Finally, providers held stereotypes and perpetuated bias related to IPV, such as thoughts that patients experiencing IPV were not reliable (Fay-Hillier, 2016; Maina, 2009; O’Doherty et al., 2016; Robinson, 2010; Sormanti & Smith, 2009; Tower et al., 2012; Zijlstra et al., 2017), were embellishing their stories or lying for their own benefit (Fay-Hillier, 2016; Loke et al., 2012; Pratt-Eriksson et al., 2014; Tower et al., 2012), were responsible for their experiences of violence (Inoue & Armitage, 2006; Robinson, 2010; Sormanti & Smith, 2009), were drug (Mayer, 2000) or attention-seeking (Fay-Hillier, 2016), were unkept or impoverished (Inoue & Armitage, 2006; Tower et al., 2012), were intoxicated (Fay-Hillier, 2016; Inoue & Armitage, 2006; Tower et al., 2012), and/or were mentally ill (Fay-Hillier, 2016).

## Discussion

### Patient Perspectives

Patients experiencing IPV reported highly variable ED care. These ranged from positive, when interacting with empathetic providers who effectively meet their needs, to negative, when interacting with judgmental providers who lacked education surrounding IPV and failed to provide necessary services.

Patients commonly described negative experiences such as feeling blamed, judged or rushed, having violence minimized, or lacking access to specialized care in the ED. Negative experiences discouraged individuals from seeking future care (Loke et al., 2012; Tower et al., 2006), likely contributing to secondary victimization and adverse health and legal outcomes (Ahrens, 2006; Campbell & Raja, 1999).

Trauma-informed care (TIC) is an approach to providing care that recognizes the widespread impact of trauma, promotes cultural safety, empowerment, and healing, and actively resists re-traumatization (ONSADVTC, 2019). Ultimately, this review demonstrated a lack of TIC contributing to decreased care-seeking behaviors. This may lead to increased morbidity and mortality, as highlighted by studies demonstrating a link between social isolation and IPV-related injury and death (Goodman & Epstein, 2008). This risk is punctuated in the context of the COVID-19 pandemic (Usher et al., 2020).

To address this, we recommend system-level change to foster TIC at the individual and institutional levels. This should include advocacy for appropriate infrastructural and financial resources within the ED, specifically focused on ensuring access to private spaces, IPV-care specialists and effective community-based programs. Of note, while universal IPV screening may provide important opportunities for intervention, it is our recommendation that appropriate resources be in place prior to universal screening implementation to avoid the harmful practice of seeing patients screen positively for IPV but not be offered appropriate support by ED providers following disclosure.

In this regard, to optimize clinical care and avoid frustration from both patients and providers, institutions require better referral systems and more highly trained teams available for around-the-clock evidence-based IPV care. While it remains an extremely complex and challenging issue, institutions also need to consider large-scale and small-scale changes to improve time constraints faced by providers. ED environments should also be adapted such that patients experiencing IPV can receive care within a space that is physically and emotionally safe.

Increased provider education is also required for TIC and we recommend providers receive training on a regular basis regarding TIC with specific applications for the ED context.

Recognizing that individual, institutional, and system issues are intertwined, initiatives such as ED-based policies and educational programs should be created in consultation with individuals holding expertise in IPV care and TIC and, whenever possible, individuals with a lived experience of IPV. These policies and programs should reflect an intersectional approach based on the unique issues providers and patients face in their specific context and should be guided by the concepts of Equity, Diversity, Inclusion, Indigeneity, and Accessibility. More details on this study’s implications for practice, policy, and research are shown in Table 3.

**Table 3.** Implications of the Review for Practice, Policy, and Research.

Level	Issue	Recommendation
Policy	Lack of provider knowledge, training, and education	● Offering longitudinal training and education on IPV, TIC, and implicit bias ● Curriculum creation should involve content-experts and individuals with lived experience to reflect patients’ needs
	Lack of institutional ability to foster TIC within ED practices and spaces	● Collaborating with content-experts and individuals with lived experience to undertake a needs assessment and implement policies/programs ● Creating easily accessible educational content addressing the different needs of patients and providers ● Creation of pathways to allow patients experiencing IPV and sexual assault to bypass waiting room ● Fostering privacy within ED (private spaces and private interview policy) ● Improving wait times
	Provider burn-out prevention	● Implementing evidence-based policies and programs to improve provider wellness ● Ensuring adequate staffing ● Fostering debriefing and improving institutional commitment to address burn-out ● Institutional recognition of vicarious trauma and investing in efforts to decrease its negative impact on providers
	Lack of access to effective and timely community and hospital-based resources for patients	● Ensuring appropriate on-call system and fostering cooperation between services for improved patient care ● Establishing link between ED and community services to improve referral systems and allow patients to have support for physical health, mental health, and legal proceedings ● Improving efficiency of referrals and management steps through clear and widely distributed policies ● Advocating for appropriate funding allocation for specialized resource offering IPV care
Practice	Lack of knowledge or comfort surrounding IPV management	● Seeking education and training to better understand the complexity of IPV; the incidence of IPV in general population and in colleagues; how to open conversations on IPV, how to respond to an IPV disclosure; legal obligations and legal outcomes ● Seeking education to understand common myths and stereotypes surrounding IPV
	Lack of TIC practice	● Adopting a TIC practice (through education and practice) ● Making a habit of counselling patients on safety planning
	Lack of knowledge of all resources	● Collaborating with content-experts, community resources and IPV-care specialists to understand available resources regarding IPV
	Lack of data on equity-deserving groups experiencing IPV	● Mandating all researchers to include diverse participant populations within recruitment and to collect detailed demographic data on gender, sexual orientation, race, ethnicity and disability
	Lack of trauma-informed methodology	● Mandating all research to detail trauma-informed methodology to Research and Ethics Board prior to project execution

EDs = emergency departments; IPV = intimate partner violence; TIC = trauma-informed care.

### Patient Care Avoidance

As a secondary objective, this review aimed to evaluate common causes of health avoidance, including why some patients decline further resources after disclosing IPV. While some patients described leaving the ED early because of fear that their partner would find them or harm them (Catallo et al., 2012; Mayer, 2000) or because of long wait times (Du Mont et al., 2013), no included studies specifically addressed this question within their primary objective. Collectively, findings suggest that patients’ perceived ability to create a better life for themselves outside of their current abusive relationship may impact decisions around accepting further intervention. Patients who felt that the healthcare system was unable to help them were less likely to invest on engaging in open discussions with providers, focusing instead on protecting themselves from re-victimization (Catallo et al., 2012, 2013; Loke et al., 2012).

When patients visited the ED, they evaluated how much they could trust a provider to avoid intrusion, which was affected by past experiences with providers and whether the present ED provider displayed a non-judgmental and supportive approach. Interacting with a trustworthy provider supported IPV disclosure. Subsequent positive interactions helped patients re-establish their self-efficacy and identity, attributes that facilitate ending violent relationships (Hosey, 2012). Conversely, when interacting with judgmental providers, patients withdrew from care.

#### Provider Perspectives

The perspectives of ED providers interacting with patients who have experienced IPV were also variable. In general, positive experiences occurred when providers had appropriate knowledge and comfort levels surrounding IPV and worked within efficient systems that were well-suited to meet patients’ needs. Conversely, negative experiences occurred when providers operated within a system that did not have adequate TIC, or when they carried personal experiences of IPV or prejudice toward their patients. Providers also reported a lack of knowledge surrounding IPV, as well as frustration and time pressures limiting their ability to appropriately manage IPV in the ED.

This SR also revealed that between one-fifth and one-third of providers have personally experienced IPV (Leppäkoski & Paavilainen, 2013; O’Malley, 2011). Many providers described the difficulty of navigating the intersection between personal and professional identities in this context, particularly when strong emotions resurfaced and made it difficult to remain professional. Recognizing how commonly IPV is experienced among providers, it is important to offer more opportunities and better tools to help providers reflect on their own trauma when caring for patients experiencing IPV.

#### Provider Stereotyping and Discrimination

Providers commonly held stereotypical beliefs toward IPV-affected patients, which led to discrimination. For example, this resulted in providers doubting the veracity of patients’ experiences of violence (Fay-Hillier, 2016; Loke et al., 2012; Maina, 2009; O’Doherty et al., 2016; Pratt-Eriksson et al., 2014; Robinson 2010; Sormanti & Smith, 2009; Tower et al., 2012; Zijlstra et al. 2017). As a result, many providers mentioned fear of liability after documenting an incident as IPV, worrying that IPV may be falsely reported by patients (Fay-Hillier, 2016; Sormanti & Smith, 2009; Zijlstra et al., 2017). The idea that individuals lie about violence or sexual assault is a myth that has repeatedly been shown to be false (Lonsway et al., 2009; Rollero, 2020). While being stereotyped certainly contributed to negative patient experiences on the individual level, these were often repetitive instances throughout patients’ lives, reflecting a pervasive systemic problem within the healthcare system.

Similarly, providers often mistrusted patients’ ability to think for themselves, perceiving them as incapable because of mental illness (Fay-Hillier, 2016), substance use (Fay-Hillier, 2016; Inoue & Armitage, 2006; Reisenhofer & Seibold, 2013; Tower et al., 2012; Yam, 2000) or because of low socio-economic status (Fay-Hillier, 2016; Inoue & Armitage, 2006; Tower et al., 2012). This likely represents a common “othering” technique used by providers as a defense mechanism to reassure oneself that they are immune to experiencing IPV (Montoya & Agustín, 2013).

In addition, patients experiencing IPV, most commonly women, were sometimes explicitly labeled by providers as “crazy” (Reisenhofer & Seibold, 2013) or “stupid” (Mayer, 2000). This reflects the “crazy woman” trope which labels woman as irrational or incompetent (Mahdawi, 2016) and often leads to victim-blaming attitudes. The gender discrimination by providers highlighted in this SR has been emphasized in previous literature (Govender & Penn-Kekana, 2008).

Many providers believed that IPV-affected patients were responsible for their experiences of violence (Inoue & Armitage, 2006; Robinson, 2010; Sormanti & Smith, 2009), an attitude known to diminish empathy toward IPV-affected individuals which has been underscored in previous studies (Garcia, 2014). In this context, providers’ duty to offer empathetic, high-quality care conflicted with negative biases held toward patients. In turn, providers adopted a paternalistic approach, lacking TIC principles and decreasing patient autonomy.

To address this, we recommend provider education initiatives focusing on information to improve provider comfort-level, debunk common myths, clarify the challenges of ending a violent relationship, and provide data regarding legal outcomes in their region. In addition, helping providers understand the impact of patients’ many intersecting social identities affecting their experiences of violence and decreasing providers’ implicit biases regarding their patients are crucial factors in improving IPV care.

#### Provider Perspective Cycle

Many providers experienced moral distress when supporting patients experiencing IPV was impossible within their imperfect system. Moral distress is defined as a phenomenon in which institutional constraints make it nearly impossible to pursue the right course of action (Widianti et al., 2019). Emergency providers were frequently disheartened when faced with the reality of inadequate services and follow-up for IPV.

Providers described a variety of emotions, from relief when a severely injured patient showed signs of improvement, to crying when managing distressing cases (van der Wath et al., 2013). They cited seeing victims who were disfigured as being particularly traumatic (Goldblatt, 2009; van der Wath et al., 2013) and explained that this affected them when they went home to their families (Goldblatt 2009; van der Wath et al., 2013), sometimes causing intrusive memories (Maina, 2009; van der Wath et al., 2013). Vicarious trauma subsequently occurred when providers were exposed to other people's trauma repeatedly (Dawson et al., 2019), a phenomenon commonly described by ED providers (Greinacher et al., 2019).

The emotional labor and burn-out associated with caring for IPV victims was discussed in a recent Cochrane SR (Rivas et al., 2019), which examined the effectiveness of advocacy interventions for women experiencing IPV. Emotional labor refers to providers being expected to regulate and suppress their emotions. Consistent with our review, emotional labor allowed for maintenance of professionalism but lent itself to provider burn-out and patients feelings dismissed.

An American study found that women experiencing IPV can make up to five attempts to leave their abuser before ending the relationship permanently (Yamawaki et al., 2012). Providers will thus often have the experience of caring multiple times for the same individuals experiencing IPV. In the face of caring for individuals who repeatedly present after a violent assault, providers described feelings of frustration and powerlessness (Colombini et al., 2013; Dawson et al., 2019; Inoue & Armitage, 2006). Specifically, providers felt frustrated when patients experiencing IPV returned to the same environment (Colombini et al., 2013; Dawson et al., 2019; Fay-Hillier, 2016; Inoue & Armitage, 2006; O’Malley, 2011; Robinson, 2010; Sormanti & Smith, 2009) or did not wish for police involvement (Fay-Hillier, 2016; Robinson, 2010; Zijlstra et al., 2017). These experiences resulted in a sense of futility and eventually resulted in many providers avoiding IPV-related discussions (Efe & Taşkın, 2012; Inoue & Armitage, 2006; Loughlin et al., 2000; Robinson, 2010; Sormanti & Smith, 2009). Some felt that this forced them to develop an impersonal approach (Sormanti & Smith, 2009; Tower et al., 2012; van der Wath et al., 2013).

These feelings of frustration and helplessness in conjunction with realization of systemic problems ultimately predispose providers to burn-out (Williston & Lafreniere, 2013). Not only does provider burn-out decrease care quality (Salyers et al., 2017), but it can also lead providers slip from understanding to blame toward their patients (Ferencik & Ramirez-Hammond, 2019).

**Figure 2.**
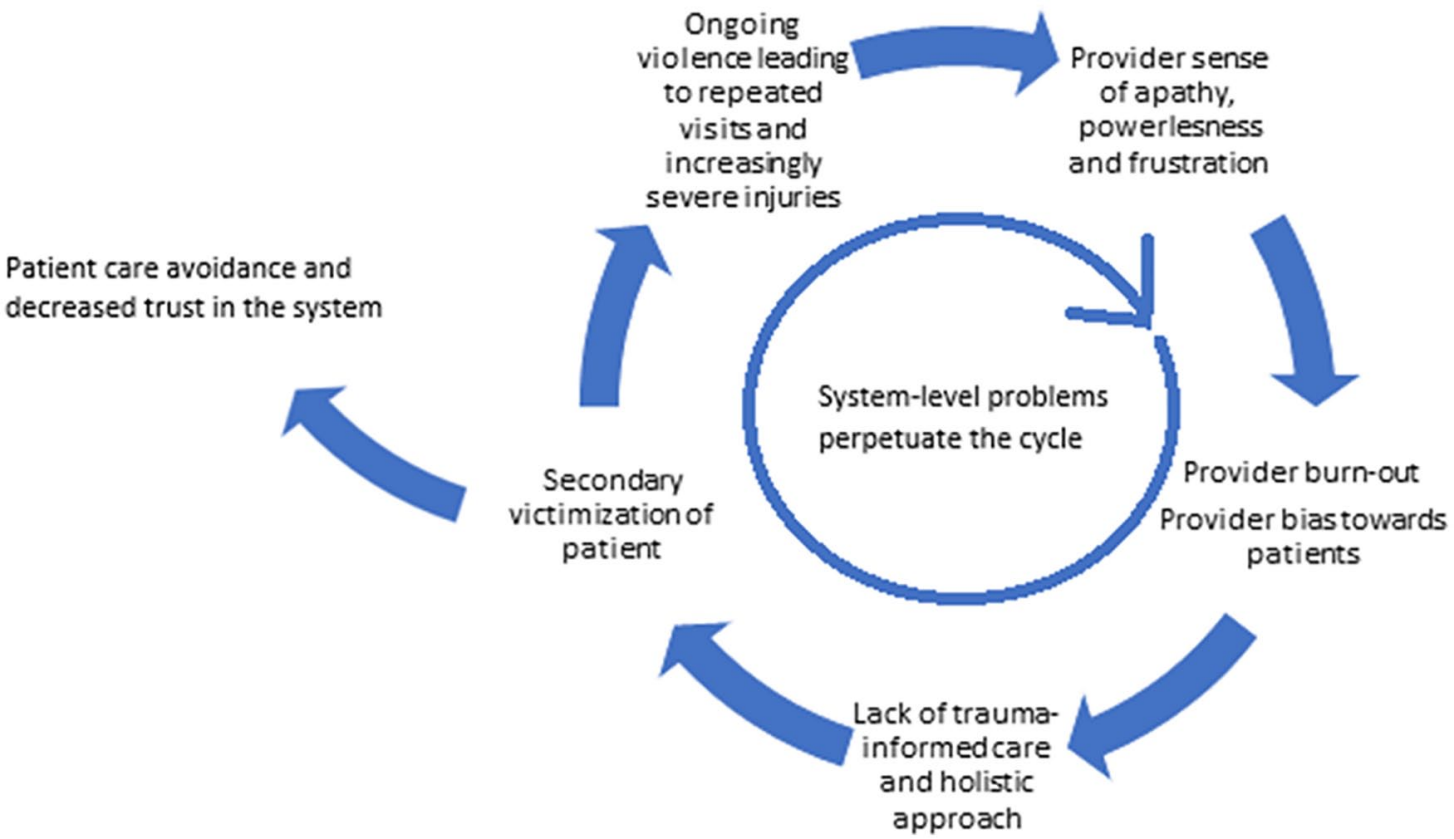
Provider perspective cycle resulting in negative patient care experiences.

In the context of ED care-seeking for IPV, patients report negative interactions when providers display harmful behaviors. This contributes to patients’ secondary victimization. Patients may approach care in a more guarded manner, potentially declining additional resources. They may also avoid ED care unless severe violence makes it absolutely necessary. These intersecting factors drawn from the literature contribute to a cycle as illustrated in Figure 2.

It is our recommendation that institutions use evidence-based interventions focused on minimizing moral distress and burn-out for ED care providers. This includes implementing comprehensive wellness initiatives, providing leadership and infrastructure that supports workers’ health and building a safe work-place culture. Finally, we recommend addressing systemic and institutional issues producing burnout such as short staffing (Mazurek, 2019).

## Limitations

### Limitations of Current Evidence

In general, the predominance of retrospective and qualitative studies using convenience sampling make this review’s results prone to bias. In addition, many studies failed to capture detailed socio-demographic data on study participants. Of the studies reporting demographic data, there was a marked lack of inclusivity of participants diverse in gender, race, ethnicity, sexual orientation, and disability in most studies leading to potential selection bias.

### Limitations of SR

The PRISMA 2009 Checklist (Moher et al., 2009) was used to reflect high-quality SR methodology. We reduced language limitation using extensive and diverse search methods, avoided outcome reporting limitations by using each author’s wording for results and independently screened, reviewed, and appraised studies to improve study rigor (Higgins & Green, 2011). However, our study does have inherent limitations. First, we may have missed some studies not published in English/French or published in other grey literature sources. Second, the World Health Organization’s IPV definition used for most studies, including ours, is incomplete because it excludes financial violence, stalking, and cyber-violence (World Health Organization, 2012) which may have under-detected participants. In addition, as is a common limitation for many studies related to IPV, patients experiencing IPV but not disclosing this would not see their experiences included in the data reviewed. Third, rigor varied within the included studies with only eight satisfying all CASP criteria and only 14 explicitly using two independent reviewers, increasing the risk of confirmation bias and decreasing validity. In addition, many studies had small sample sizes with 81% of studies having less than 50 participants. While an appropriate sample size in qualitative research is still debated (Vasileiou et al., 2018), saturation was only reported in 14 studies. While we included studies regardless of quality to ensure no data were missed, this introduces challenges in interpreting results from the studies with limited internal validity.

## Strengths

This SR adds to the current body of knowledge and extends beyond previous reviews since it is the first to synthesize study findings associated with negative and positive care experiences from both patient and provider perspectives. It also uniquely undertook a detailed analysis of demographic and social identity data recorded in each study and specifically used search strategies aiming to include perspectives from a diverse range of patients and providers.

This review holistically summarizes ED-specific contributors to positive and negative care experiences. There are few existing SRs exploring ED IPV-care experiences, with most focus on IPV-screening efficacy. While perspectives from nurses and physicians continue to have the strongest presence in the literature, our SR successfully included evidence from a wider variety of provider backgrounds.

### Recommendations for Future Research

More studies evaluating the impact of negative ED experiences on overall access to care and ability to stop, avoid, or escape violence are needed. Further studies focusing on ED care experiences after a remote experience of IPV and on follow-up care offered through EDs would be of benefit. Several studies captured the provider burden of caring for IPV-affected patients—another area requiring further research.

This review revealed large gaps in the literature surrounding patients and providers who identify as male or as members of equity-deserving groups such as Two-Spirit, lesbian, gay, bisexual, transgender, queer or questioning, BIPOC or experiencing a disability. Pointed efforts are necessary to include a diverse range of ED patient and provider perspectives. Detailed participant demographic data surrounding age, gender, sexual orientation, race, ethnicity, language, and disability should be prioritized.

## Conclusion

This SR of ED care experiences for patients with lived experience of IPV and their providers identified contributors to positive experiences including access to private and timely care, knowledgeable providers, and accessible specialized care teams with efficient referral systems. Primary contributors to negative care experiences included provider lack of knowledge and prejudice, lack of trauma-informed and patient-centered care, lack of time and privacy in the ED, and lack of accessible IPV-care teams. This review also indicated that both parties experience frustration and hopelessness associated with the limitations of the healthcare system, stemming from complex interactions at the individual, institutional, and systemic levels. Findings underscore the need for increased attention toward more inclusive population recruitment in future studies. Correcting the systemic, institutional, and individual problems outlined in this review should be guided by intersectional, equity-based policy creation, as well as promotion of provider education and burn-out prevention. The findings of this SR are a step toward elucidating research needs and interventions to provide high-quality, TIC that will contribute to reduced morbidity and mortality related to IPV.

## Supplemental Material

sj-docx-1-tva-10.1177_15248380221118962 – Supplemental material for Patient and Provider Emergency Care Experiences Related to Intimate Partner Violence: A Systematic Review of the Existing Evidence Click here for additional data file. Supplemental material, sj-docx-1-tva-10.1177_15248380221118962 for Patient and Provider Emergency Care Experiences Related to Intimate Partner Violence: A Systematic Review of the Existing Evidence by Emma Duchesne, Aisha Nathoo, Melanie Walker and Susan A. Bartels in Trauma, Violence, & Abuse
