# Supplementary material for: Patient and Provider Emergency Care Experiences Related to Intimate Partner Violence: A Systematic Review of the Existing Evidence
Source: Trauma Violence Abuse. 2022 Aug 23;24(5):2901–21. doi: 10.1177/15248380221118962 (PMC10594849; doi:10.1177/15248380221118962)
Supplement: sj-docx-1-tva-10.1177_15248380221118962 – Supplemental material for Patient and Provider Emergency Care Experiences Related to Intimate Partner Violence: A Systematic Review of the Existing Evidence [file sj-docx-1-tva-10.1177_15248380221118962.docx]

**Appendix**

**Appendix A - MeSH/Keywords Search Strategy**

| **Name of database** | **MeSH terms used** |
| --- | --- |
| **Embase** | partner violence/ or domestic violence/ or marital rape/ or battered woman/  AND  personal experience/ or attitude/ Or exp professional patient relationship/ or perspective.mp. Or perspectives.pm. Or stories.mp. Or story.mp. Or interactions.mp. Or interaction.mp.  AND  emergency ward/ or emergency medicine/ or rescue personnel/ |
| **Cochrane EBM Reviews - Cochrane Central Register of Controlled Trials** | Spousal abuse/  AND  Attitude/ or perspective.mp. Or perspectives.mp. Or story.mp. Or Stories.mp. Or Experience.mp. Or experiences.mp. Or interaction.mp. Or interactions.mp.  or physician-patient relations/ |
| **Cochrane EBM Reviews - Cochrane Central Register of Controlled Trials** | Spousal abuse/  AND  Attitude/ or perspective.mp. Or perspectives.mp. Or story.mp. Or Stories.mp. Or Experience.mp. Or experiences.mp. Or interaction.mp. Or interactions.mp.  or physician-patient relations/  AND  Emergency Medicine/ or Emergency Service, Hospital |
| **Cochrane EBM Reviews – Cochrane Database of Systematic Reviews** | **Search 1**  partner violence.mp. [mp=title, short title, abstract, full text, keywords, caption text] Or intimate partner violence.mp. [mp=title, short title, abstract, full text, keywords, caption text] Or spousal abuse.mp. [mp=title, short title, abstract, full text, keywords, caption text] Or marital rape.mp. [mp=title, short title, abstract, full text, keywords, caption text] Or battered woman.mp. [mp=title, short title, abstract, full text, keywords, caption text] Or battered wife.mp. [mp=title, short title, abstract, full text, keywords, caption text]  AND  experiences.mp. [mp=title, short title, abstract, full text, keywords, caption text] Or  perspectives.mp. [mp=title, short title, abstract, full text, keywords, caption text] Or experience.mp. [mp=title, short title, abstract, full text, keywords, caption text] Or perspective.mp. [mp=title, short title, abstract, full text, keywords, caption text] Or attitude.mp. [mp=title, short title, abstract, full text, keywords, caption text] Or attitudes.mp. [mp=title, short title, abstract, full text, keywords, caption text] Or story.mp. [mp=title, short title, abstract, full text, keywords, caption text] Or stories.mp. [mp=title, short title, abstract, full text, keywords, caption text] Or patient-doctor relationship.mp. [mp=title, short title, abstract, full text, keywords, caption text] Or physician-patient relationship.mp. [mp=title, short title, abstract, full text, keywords, caption text] Or doctor-patient relationship.mp. [mp=title, short title, abstract, full text, keywords, caption text] Or interaction.mp. [mp=title, short title, abstract, full text, keywords, caption text] Or Interactions.mp. [mp=title, short title, abstract, full text, keywords, caption text]  **Search 2**  partner violence.mp. [mp=title, short title, abstract, full text, keywords, caption text] Or intimate partner violence.mp. [mp=title, short title, abstract, full text, keywords, caption text] Or spousal abuse.mp. [mp=title, short title, abstract, full text, keywords, caption text] Or marital rape.mp. [mp=title, short title, abstract, full text, keywords, caption text] Or battered woman.mp. [mp=title, short title, abstract, full text, keywords, caption text] Or battered wife.mp. [mp=title, short title, abstract, full text, keywords, caption text]  AND  experiences.mp. [mp=title, short title, abstract, full text, keywords, caption text] Or  perspectives.mp. [mp=title, short title, abstract, full text, keywords, caption text] Or experience.mp. [mp=title, short title, abstract, full text, keywords, caption text] Or perspective.mp. [mp=title, short title, abstract, full text, keywords, caption text] Or attitude.mp. [mp=title, short title, abstract, full text, keywords, caption text] Or attitudes.mp. [mp=title, short title, abstract, full text, keywords, caption text] Or story.mp. [mp=title, short title, abstract, full text, keywords, caption text] Or stories.mp. [mp=title, short title, abstract, full text, keywords, caption text] Or patient-doctor relationship.mp. [mp=title, short title, abstract, full text, keywords, caption text] Or physician-patient relationship.mp. [mp=title, short title, abstract, full text, keywords, caption text] Or doctor-patient relationship.mp. [mp=title, short title, abstract, full text, keywords, caption text] Or interaction.mp. [mp=title, short title, abstract, full text, keywords, caption text] Or Interactions.mp. [mp=title, short title, abstract, full text, keywords, caption text]  AND  emergency department.mp. [mp=title, short title, abstract, full text, keywords, caption text] Or emergency ward.mp. [mp=title, short title, abstract, full text, keywords, caption text] Or paramedical service.mp. [mp=title, short title, abstract, full text, keywords, caption text] Or emergency medical transport.mp. [mp=title, short title, abstract, full text, keywords, caption text] Or paramedic.mp. [mp=title, short title, abstract, full text, keywords, caption text] Or Rescue Personnel.mp. [mp=title, short title, abstract, full text, keywords, caption text] |
| **Medline (Ovid)** | Domestic violence or spouse abuse or intimate partner violence or battered women  AND  Attitude of health personnel or professional-patient relations or nurse-patient relations or physician-patient relations or * experience or *perspective or *attitude or *interaction or *story or *stories  AND  Emergency medical services or emergency service, hospital |
| **PsychINFO (Ovid)** | Intimate partner violence  AND  Health personnel attitudes or *experience or *perspective or *attitude or *interaction or *story or *stories  AND  Emergency medicine or emergency services or emergency personnel or paramedics |
| **Google Scholar** | **Search 1**intimate partner violence + emergency department + (experience OR attitude)  **Search 2**  intimate partner violence + emergency services + (experience OR attitude)  **Search 3**Spouse abuse + emergency department + (experience OR attitude)  **Search 4**Spouse abuse + emergency services+ (experience OR attitude)  **Search 5**Domestic violence + emergency services+ (experience OR attitude)  **Search 6**Domestic violence + emergency department + (experience OR attitude) |
| **CINAHL and Pre-CINAHL** | Emergency Medicine OR Emergency Service OR Emergency Medical Technician Attitudes OR Physicians, Emergency  AND  Intimate Partner Violence OR Domestic Violence  AND  Life Experiences or *Experiences |
| **Proquest** | *Experiences  AND  *Intimate Partner Violence  AND  *Emergency Department  *Source type: blogs, podcasts, websites, conference papers & proceedings, dissertations & theses* ; *Language: English, French* |

**Appendix B - Snowballing Search Strategy**

| **Article** | **Relevant articles imported** |
| --- | --- |
| Tower, M., Rowe, J., & Wallis, M. (2012). Reconceptualising health and health care for women affected by domestic violence. Contemporary nurse, 42(2), 216–225. <https://doi.org/10.5172/conu.2012.42.2.216> | 1 |
| Ahmad, I., Ali, P. A., Rehman, S., Talpur, A., & Dhingra, K. (2017). Intimate partner violence screening in emergency department: a rapid review of the literature. Journal of clinical nursing, 26(21-22), 3271–3285. <https://doi.org/10.1111/jocn.13706> | 20 |
| Bakon, S., Taylor, A., Meyer, S., & Scott, M. (2019). The provision of emergency healthcare for women who experience intimate partner violence: part 1. An integrative review. Emergency nurse : the journal of the RCN Accident and Emergency Nursing Association, 27(6), 19–25. <https://doi.org/10.7748/en.2019.e1950> | 11 |
| Bakon, S., Taylor, A., Meyer, S., & Scott, M. (2020). The provision of emergency healthcare for women who experience intimate partner violence: part 2. Strategies to address knowledge deficits and negative attitudes. Emergency nurse : the journal of the RCN Accident and Emergency Nursing Association, 28(4), 18–23. <https://doi.org/10.7748/en.2020.e1994> | 16 |
| Hinsliff-Smith, K., & McGarry, J. (2017). Understanding management and support for domestic violence and abuse within emergency departments: A systematic literature review from 2000-2015. Journal of clinical nursing, 26(23-24), 4013–4027. <https://doi.org/10.1111/jocn.13849> | 24 |
| Chung, M. Y., Wong, T. W., & Yiu, J. J. (1996). Wife battering in Hong Kong: accident and emergency nurses' attitudes and beliefs. Accident and emergency nursing, 4(3), 152–155. <https://doi.org/10.1016/s0965-2302(96)90063-6> | 10 |
| Mayer B. W. (2000). Female domestic violence victims: perspectives on emergency care. Nursing science quarterly, 13(4), 340–346. <https://doi.org/10.1177/089431840001300419> | 8 |
| Williston, C. J., & Lafreniere, K. D. (2013). "Holy cow, does that ever open up a can of worms": health care providers' experiences of inquiring about intimate partner violence. Health care for women international, 34(9), 814–831. <https://doi.org/10.1080/07399332.2013.794460> | 24 |
| Dawson, A. J., Rossiter, C., Doab, A., Romero, B., Fitzpatrick, L., & Fry, M. (2019). The Emergency Department Response to Women Experiencing Intimate Partner Violence: Insights From Interviews With Clinicians in Australia. Academic emergency medicine : official journal of the Society for Academic Emergency Medicine, 26(9), 1052–1062. <https://doi.org/10.1111/acem.13721> | 23 |
| Yam M. (2000). Seen but not heard: battered women's perceptions of the ED experience. Journal of emergency nursing, 26(5), 464–470. <https://doi.org/10.1067/men.2000.110432> | 8 |
| McGrath, M. E., Bettacchi, A., Duffy, S. J., Peipert, J. F., Becker, B. M., & St Angelo, L. (1997). Violence against women: provider barriers to intervention in emergency departments. Academic emergency medicine : official journal of the Society for Academic Emergency Medicine, 4(4), 297–300. <https://doi.org/10.1111/j.1553-2712.1997.tb03552.x> | 3 |
| Zijlstra, E., van de Laar, R., Moors, M. L., Lo Fo Wong, S., & Lagro-Janssen, A. (2017). Tensions and Ambivalent Feelings: Opinions of Emergency Department Employees About the Identification and Management of Intimate Partner Violence. Journal of interpersonal violence, 32(7), 1044–1067. <https://doi.org/10.1177/0886260515587663> | 27 |
| Cho, O. H., Cha, K. S., & Yoo, Y. S. (2015). Awareness and Attitudes Towards Violence and Abuse among Emergency Nurses. Asian nursing research, 9(3), 213–218. <https://doi.org/10.1016/j.anr.2015.03.003> | 9 |
| Garimella, R., Plichta, S. B., Houseman, C., & Garzon, L. (2000). Physician beliefs about victims of spouse abuse and about the physician role. Journal of women's health & gender-based medicine, 9(4), 405–411. <https://doi.org/10.1089/15246090050020727> | 9 |
